# Safety and Efficacy of Low-Pressure Pneumoperitoneum in Laparoscopic Cholecystectomy: A Prospective Observational Study

**DOI:** 10.7759/cureus.97898

**Published:** 2025-11-26

**Authors:** Debiprasad Sahoo, Harsh J Barot, Nitin Borle

**Affiliations:** 1 General Surgery, Topiwala National Medical College and B. Y. L. Nair Charitable Hospital, Mumbai, IND

**Keywords:** general surgery minimal access surgery laparoscopic surgery, lap chole, minimal invasive approach, pneumoperitoneum, postoperative pain relief

## Abstract

Introduction: Conventional laparoscopic cholecystectomy uses pneumoperitoneum pressures of 12-15 mmHg, which may contribute to postoperative pain and physiological disturbances. This study evaluates the safety and efficacy of low-pressure pneumoperitoneum (LPP, 7-10 mmHg) in laparoscopic cholecystectomy.

Materials and methods: In this prospective observational study conducted at a tertiary care center, 70 patients undergoing elective laparoscopic cholecystectomy were enrolled. Patients were operated on using LPP; conversions to standard-pressure pneumoperitoneum (SPP) or open surgery were recorded along with intraoperative and postoperative outcomes. Pain scores, operative time, complications, hemodynamics, and hospital stay were assessed.

Results: The mean age was 40.02 years with female predominance (78.6%). Conversion to SPP occurred in patients with higher mean arterial pressures and larger common bile duct diameters. The 10 mmHg group had significantly shorter operative times (mean: 79.6 min) than the 12 mmHg (113.1 min) and 14 mmHg (140 min) groups. Postoperative pain scores were significantly lower in the LPP group across all time intervals. The majority of patients (64.3%) were discharged within 24 hours. No significant complications or mortality occurred.

Conclusions: LPP is a safe and effective approach in laparoscopic cholecystectomy, associated with shorter operative times, reduced pain, and faster recovery. It can be considered as a feasible alternative to SPP in appropriately selected patients.

## Introduction

Laparoscopic cholecystectomy (LC) is the gold standard treatment for symptomatic cholelithiasis due to its clear advantages over open surgery, including reduced postoperative pain, shorter hospital stays, and quicker recovery [1]. Despite the widespread adoption of this minimally invasive approach, the physiological impact of pneumoperitoneum, particularly elevated intra-abdominal pressure (IAP), has drawn increasing scrutiny. The standard insufflation pressure during laparoscopic cholecystectomy is typically set at 12-15 mmHg, a range considered optimal for adequate visualization and operative space. However, sustained exposure to high IAP has been associated with adverse effects on pulmonary mechanics, cardiovascular performance, renal function, hepatic perfusion, and overall hemodynamic stability [2-4].

The advent of automatic insufflation devices in the 1960s, particularly the work of Kurt Semm, revolutionized laparoscopic surgery by enabling precise control of IAP [3]. This technological advancement highlighted the systemic effects of pneumoperitoneum, prompting ongoing research to optimize pressure settings to improve safety and patient outcomes.

Emerging evidence supports the use of low-pressure pneumoperitoneum (LPP), generally defined as an IAP of 7-10 mmHg, as a safer alternative to conventional pressures [5]. LPP may reduce the physiological burden on organ systems, minimize postoperative pain, and enhance recovery, while still providing acceptable surgical exposure [6]. Nevertheless, concerns remain regarding its impact on operative field visibility, procedure duration, complication rates, and the risk of conversion to high-pressure pneumoperitoneum (HPP) or open surgery [7].

This study aims to evaluate the safety, feasibility, and clinical outcomes associated with LPP in patients undergoing laparoscopic cholecystectomy. Key outcome measures include operative time, visualization quality, gas consumption, conversion rates, postoperative pain, complication rates, recovery profiles, and hospital stay duration. By comparing these parameters with conventional pressure standards, we aim to provide meaningful evidence to optimize pneumoperitoneum settings in routine laparoscopic practice [8,9].

## Materials and methods

Study design and setting

This was a prospective observational study conducted at a tertiary care hospital in Mumbai over 24 months. Ethical clearance was obtained from the Ethics Committee for Academic Research Projects (ECARP), PG Academic Committee, T.N. Medical College, and BYL Nair Charitable Hospital (approval number: ECARP/2023/09). Written informed consent was obtained from all study participants before enrollment.

Study population

A total of 70 adult patients undergoing elective laparoscopic cholecystectomy for symptomatic cholelithiasis were included in the study. The study population was recruited consecutively from protocol approval through August 2024.

Inclusion and exclusion criteria

Patients undergoing laparoscopic cholecystectomy for confirmed symptomatic cholelithiasis between April 2024 and August 2024 in the Department of General Surgery who provided written informed consent were included in the study. Patients with gallbladder malignancy, those diagnosed with acute cholecystitis, and those requiring a pneumoperitoneum pressure greater than 10 mmHg from the start of surgery were excluded. The sample size of 61 patients was estimated based on the average monthly number of admissions for symptomatic cholelithiasis at our institution, and to account for potential dropouts, a total of 70 patients were enrolled in the study.

Study design

All patients underwent laparoscopic cholecystectomy under general anesthesia, following a standardized operative protocol: the four-port technique (one infraumbilical 10 mm port for the camera, one epigastric 10 mm port, and two 5 mm ports for working and retraction). A critical view of safety was achieved in every case before clipping the cystic artery and cystic duct. Pneumoperitoneum was created using carbon dioxide, and IAP was maintained between 7 and 10 mmHg. Any patient requiring pressure >10 mmHg from the beginning was excluded. If, at any stage during the operation, the surgeon encountered inadequate visualization or other surgical difficulties, the insufflation pressure could be increased to SPP (12-15 mmHg), or the procedure could be converted to an open cholecystectomy as deemed necessary. Reasons for conversion were documented and included factors such as deranged vitals, intraoperative complications, or equipment failure. Blood pressure and pulse rate were recorded at defined intervals: preoperatively, immediately after intubation, before pneumoperitoneum, every 20 minutes intraoperatively, and immediately after pneumoperitoneum release. Closure of ports followed standard protocol. The same surgeon performed all surgeries to strengthen the methodology.

Postoperative management

Postoperative vital signs were monitored at six hours postoperatively and then every 12 hours during the hospital stay. Pain was managed with a round-the-clock analgesic regimen comprising intravenous paracetamol 1 g every six hours, intravenous diclofenac 50 mg every 12 hours, and intravenous tramadol 50 mg every 12 hours for up to 36 hours post-surgery in all patients (including those with HPP and LPP). Patients were allowed sips of water at six hours postoperative and progressed to a liquid diet at 12 hours if tolerated. A regular diet was introduced at 24 hours postoperative upon the return of bowel sounds. Early ambulation was encouraged. Any complications were managed as per standard clinical guidelines, and discharge was delayed accordingly.

Data collection

Data were collected using a pre-designed case record form and entered into Microsoft Excel (Microsoft Corp., Redmond, WA, USA). Variables included demographic details, clinical findings, intraoperative parameters (e.g., duration, conversion rate, gas consumption), and postoperative outcomes (e.g., pain, complications, recovery time, and hospital stay). There were no conversions from laparoscopic to open. Preoperative ultrasonography showed a normal CBD size.

Statistical analysis

Data analysis was performed using SPSS Statistics version 25.0 (IBM Corp. Released 2017. IBM SPSS Statistics for Windows, Version 25.0. Armonk, NY: IBM Corp.). Quantitative variables were expressed as means ± standard deviations or medians with interquartile ranges, depending on the distribution. Categorical variables were summarized as frequencies and percentages.

Normality was assessed using the Kolmogorov-Smirnov and Shapiro-Wilk tests, which revealed non-normal distribution for all continuous variables (p<0.001). Accordingly, nonparametric tests such as the Mann-Whitney U test and the Kruskal-Wallis test were used to compare continuous variables. For categorical data, chi-square or Fisher’s exact tests were applied. A p-value of <0.05 was considered statistically significant. Graphical representations such as bar charts and pie diagrams were used to enhance data visualization.

## Results

A total of 70 patients undergoing laparoscopic cholecystectomy were analyzed in this study. None of the patients required HPP from the start of the operation. Table 1 describes baseline characteristics. BMI was less than 24 kg/m² in all patients; none were obese.

**Table 1:** Baseline characteristics of study participants in the LPP and HPP groups † p-values computed using an independent t-test for continuous variables and chi-square or Fisher’s exact test for categorical variables. Values presented as mean ± SD or n (%). SD: standard deviation, LPP: low-pressure pneumoperitoneum, HPP: high-pressure pneumoperitoneum

Characteristic	LPP group (10 mmHg) (n=40)	HPP group (12–14 mmHg) (n=30)	p-value †
Age (years)	44.6 ± 8.7	46.3 ± 9.1	0.42
Sex			
Female	32 (80.0%)	23 (76.7%)	0.72
Male	8 (20.0%)	7 (23.3%)	
Comorbidities			
Diabetes mellitus	10 (25.0%)	8 (26.7%)	0.86
Hypertension	9 (22.5%)	6 (20.0%)	0.78
Ischemic heart disease	1 (2.5%)	1 (3.3%)	0.82
COPD/asthma	1 (2.5%)	0 (0.0%)	0.39

A total of 30 patients (42.9%) required intraoperative conversion from LPP to HPP during laparoscopic cholecystectomy. The most frequent causes were poor visualization or restricted working space (30%) and intraoperative elevation of mean arterial pressure (MAP; 26.7%), both of which were cited as primary reasons for conversion in operative records.

Other contributing factors included dense adhesions or difficult dissection (16.7%), bile or stone spillage (10%), and a dilated common bile duct (10%). Conversion due to gallbladder tear and machine malfunction was rare (3.3% each). These findings highlight that hemodynamic instability and limited intraoperative visibility remain the main drivers for conversion from LPP to HPP during laparoscopic cholecystectomy. Table 2 describes the conversion data.

**Table 2:** Reasons for conversion from LPP to HPP during laparoscopic cholecystectomy MAP: mean arterial pressure, CBD: common bile duct, LPP: low-pressure pneumoperitoneum, HPP: high-pressure pneumoperitoneum

Reason for conversion	Number of patients (n)	% of conversions (n=30)	% of total cohort (n=70)
Elevated intraoperative MAP (rise >20% from baseline)	8	26.70%	11.40%
Poor visualization/restricted working space	9	30.00%	12.90%
Dense adhesions/difficult dissection	5	16.70%	7.10%
Spillage of bile or stone requiring higher pressure	3	10.00%	4.30%
Large CBD size/dilated biliary tree	3	10.00%	4.30%
Gallbladder wall tear/intraoperative injury	1	3.30%	1.40%
Machine malfunction/gas leak	1	3.30%	1.40%
Total conversions	30	100%	42.90%

Postoperative pain scores were consistently lower in the LPP group than in the HPP at all measured time points. At six hours, the median pain score was 4 (IQR 4-6) in the LPP group versus 6 (IQR 6-8) in the HPP group (p=0.012). At 12 hours, pain decreased further to 3 (IQR 2-4) and 5 (IQR 4-6), respectively (p=0.005). By 24 hours, the median pain score was 2 (IQR 2-3) in the LPP group, compared with 4 (IQR 3-5) in the HPP group (p<0.001). These findings demonstrate a clear trend of reduced postoperative pain with LPP, with the difference becoming more pronounced over time. Table 3 describes pain scores.

**Table 3:** Comparison of median postoperative pain scores between the LPP and HPP groups † Kruskal–Wallis test with post-hoc Dunn’s pairwise comparison (Holm correction). * p<0.05, ** p<0.01, *** p<0.001. IQR: interquartile range, LPP: low-pressure pneumoperitoneum, HPP: high-pressure pneumoperitoneum

Time after surgery	LPP group (10 mmHg) - median (IQR)	HPP group (12-14 mmHg) - median (IQR)	p-value†
6 hours	4 (4-6)	6 (6-8)	0.012*
12 hours	3 (2-4)	5 (4-6)	0.005**
24 hours	2 (2-3)	4 (3-5)	<0.001***

Intraoperative MAP was significantly lower in the LPP group at 30, 60, and 90 minutes (all p<0.05), indicating a more stable hemodynamic profile with reduced cardiopulmonary strain. Postoperative recovery of bowel function was faster in the LPP group, with audible bowel sounds on postoperative day 1 in 85% of patients compared to 60% in the HPP group (p=0.021). By day 2, almost all patients in the LPP group had recovered bowel sounds, while delayed recovery persisted in 40% of the HPP group (p=0.018).

The incidence of bile or stone spillage and postoperative bile leak was low in both groups, with no statistically significant difference (p>0.05). Postoperative bile leaks were observed in two patients (2.9%), both from the HPP group, which was treated with endoscopic retrograde cholangiopancreatography (ERCP) and CBD stenting. Table 4 describes the secondary outcomes among both groups.

**Table 4:** Comparison of secondary outcomes between the LPP and HPP groups † p-values calculated using independent t-test or Mann-Whitney U test for continuous variables and chi-square/Fisher’s exact test for categorical variables. Values are presented as mean ± SD or n (%). * p<0.05, ** p<0.01. MAP: mean arterial pressure, SD: standard deviation, POD: postoperative day, LPP: low-pressure pneumoperitoneum, HPP: high-pressure pneumoperitoneum

Secondary outcome	LPP group (10 mmHg) (n=40)	HPP group (12-14 mmHg) (n=30)	p-value†
Intraoperative MAP (mmHg)			
30 min	84.3 ± 5.6	91.5 ± 6.9	0.008**
60 min	86.0 ± 5.6	94.1 ± 6.3	0.010*
90 min	87.2 ± 6.3	99.1 ± 7.8	0.049*
Bowel sounds present on POD 1	34 (85.0%)	18 (60.0%)	0.021*
Bowel sounds present on POD 2	6 (15.0%)	12 (40.0%)	0.018*
Conversion to higher pressure	-	5 (16.7%)	-
Bile or stone spillage	2 (5.0%)	3 (10.0%)	0.42
Postoperative bile leak	0 (0.0%)	2 (6.7%)	0.14

Collectively, these findings suggest that LPP is associated with better intraoperative hemodynamic stability and faster postoperative recovery of bowel function, without increasing operative complications.

## Discussion

This study evaluated the clinical outcomes of patients undergoing laparoscopic cholecystectomy under LPP (10 mmHg) and HPP (12-14 mmHg), focusing on demographic characteristics, operative parameters, postoperative outcomes, and predictors of conversion. The findings provide valuable insights into the efficacy and safety of LPP in routine surgical practice.

Demographic profile

A total of 70 patients were analyzed; none required HPP at the start of surgery. The majority of patients were female, consistent with the higher prevalence of gallstone disease among women. The most common age group was 46-50 years, with a mean age in the mid-40s, similar to that reported by Thapa et al. [10] and Abdallah et al. [11]. These findings reinforce the demographic consistency across populations undergoing laparoscopic cholecystectomy. As none of the patients were obese, obesity as a factor in the difficulty of the procedure in an LPP setting remains to be assessed.

Postoperative pain and analgesic requirements

Pain assessment across the first 24 hours postoperatively demonstrated that patients in the LPP group consistently experienced less pain than those in the HPP group. Median pain scores were significantly lower at six hours (p=0.012), 12 hours (p=0.005), and 24 hours (p<0.001). These results support the hypothesis that reduced IAP decreases peritoneal stretch and diaphragmatic irritation, thereby improving postoperative comfort. Recent studies corroborate these findings. Similarly, Thapa et al. [10], Ali et al. [12], and Meena et al. [13] reported lower VAS scores and lower analgesic requirements among patients operated under LPP. Collectively, these studies confirm the analgesic and recovery benefits of LPP. Rashdan et al. [14] reported significantly lower postoperative pain and lower inflammatory marker levels in the LPP group than in the SPP group. Rosenberg [15] emphasized that LPP reduces postoperative pain and analgesic requirements without increasing complications.

Hospital stay and recovery

Most patients were discharged within 24-48 hours, underscoring the minimally invasive nature of laparoscopic cholecystectomy. The LPP group had a shorter mean hospital stay (1.1 ± 0.3 days) compared with the HPP group (1.4 ± 0.5 days; p=0.018), indicating faster recovery. These observations are consistent with Thapa et al. [10], Abdallah et al. [11], and Meena et al. [13], all of whom reported shorter postoperative hospitalization with LPP. The evidence supports that LPP facilitates earlier discharge and enhances postoperative recovery.

Operative time and intraoperative parameters

The mean operative time was significantly longer in the LPP group (79.6 ± 17.0 min) than in the HPP group (118.2 ± 24.6 min; p<0.001). The difference is attributed to the reduced working space and limited visualization at LPP. Despite this, the intraoperative complication rate remained low, confirming the technical feasibility and safety of LPP. A total of 30 patients (42.9%) required intraoperative conversion to HPP. The leading causes were poor visualization (30%) and elevated intraoperative MAP (26.7%), followed by dense adhesions (16.7%), bile or stone spillage (10%), and CBD dilatation (10%). These findings are consistent with prior studies that identified inadequate exposure and hemodynamic instability as major drivers for conversion [7,11].

Tian et al. [16] demonstrated that LPP at 10 mmHg is feasible even in patients with cardiopulmonary comorbidities, while Koç et al. [17] found that combining LPP with deep neuromuscular blockade improved operative exposure without increasing complications. Together, these studies reinforce that LPP laparoscopic cholecystectomy is safe and adaptable across patient profiles.

Intraoperative hemodynamics and physiological impact

Intraoperative MAP was significantly lower in the LPP group at 30, 60, and 90 minutes (all p<0.05), indicating reduced cardiovascular strain and better hemodynamic stability. These results parallel findings by Abdallah et al. [11] and Meena et al. [13]. Postoperative bowel function also returned more rapidly under LPP, with 85% of patients demonstrating bowel sounds by postoperative day 1 compared to 60% in the HPP group (p=0.021). Park et al. [18] further demonstrated that even extremely LPP is feasible and safe without increasing complications. Collectively, these data suggest that LPP not only stabilizes intraoperative physiology but also promotes faster postoperative recovery.

Predictors of conversion

Regression analysis identified higher intraoperative MAP and larger CBD diameter as independent predictors of conversion to HPP. These factors likely indicate hemodynamic intolerance and altered biliary anatomy, which may compromise operative visibility under LPP. Conversely, bile or stone spillage and gallbladder tear were not significant predictors, suggesting that such intraoperative events alone do not necessitate conversion. Similar observations have been reported by Bhattacharjee et al. [7], who also identified complex anatomy and physiological instability as the leading causes of an escalation in pneumoperitoneum pressure.

Postoperative nausea and biliary complications

A significant association was observed between bile or stone spillage and postoperative nausea, aligning with the findings of Bhattacharjee et al. [7]. This underscores the importance of careful dissection and retrieval techniques to reduce peritoneal contamination. Postoperative bile leaks were rare and occurred exclusively in the HPP group (2.9%), both successfully managed with ERCP and CBD stenting. Similar trends have been noted by Abdallah et al. [11] and Meena et al. [13], further supporting the safety advantage of LPP.

Visualization changes in LPP compared to SPP

**Figure 1:**
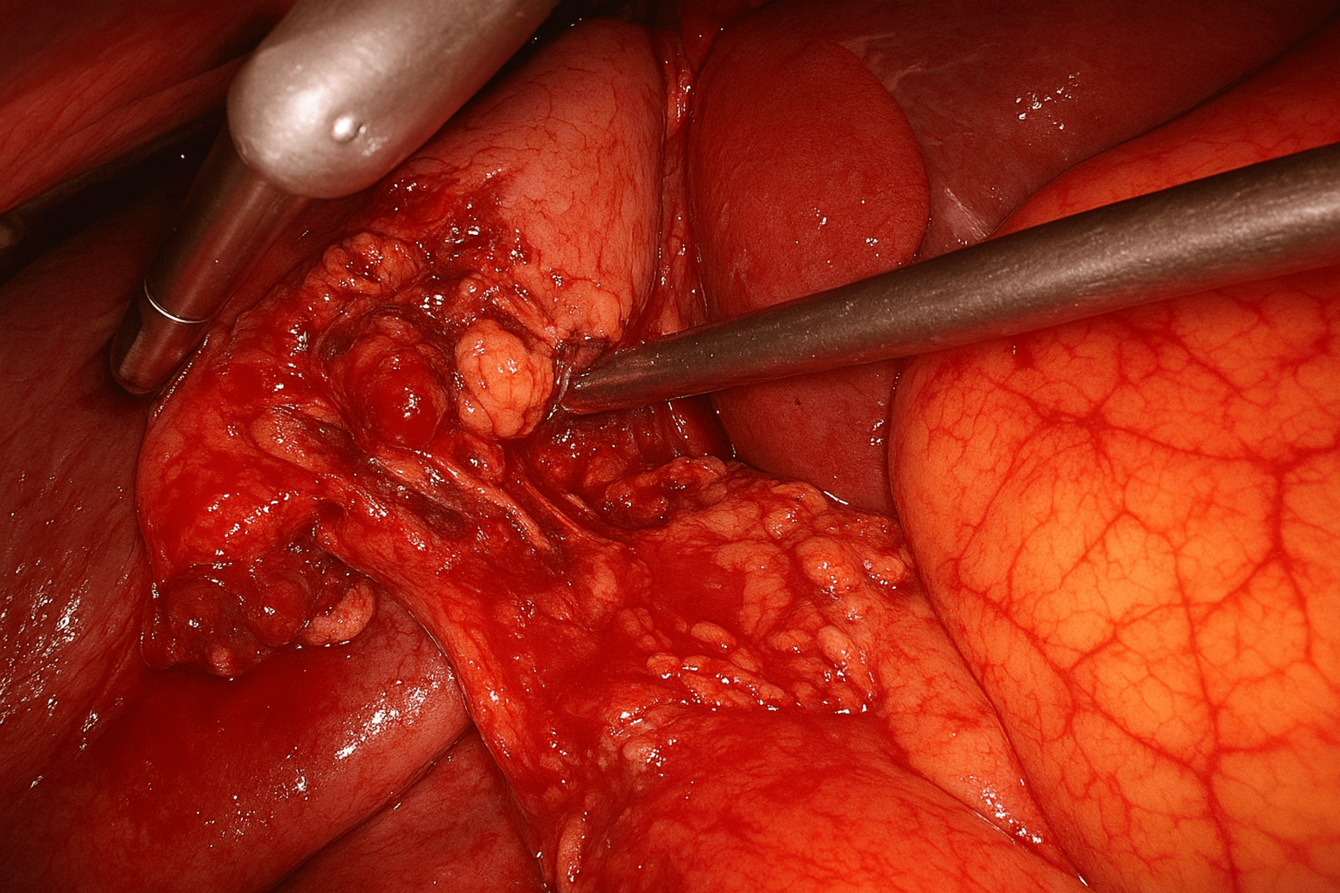
Intraoperative image of LPP laparoscopic cholecystectomy LPP: low-pressure pneumoperitoneum

In LPP, the reduced abdominal distension (typically 6-8 mmHg) results in a noticeably narrower operative field, as seen in an intraoperative photograph (Figure 1). The tissues lie closer together, so the hepatocystic triangle appears more crowded, with less separation between the gallbladder, liver edge, and surrounding fat. Retraction provides less lift, making the critical view harder to expose. Smoke clearance is slower, and minor oozing tends to obscure the field more quickly because there is less space for gas circulation. Overall, visualization is feasible but tighter and less panoramic compared to the wide, fully expanded field at SPP (12-14 mmHg).

Study limitations

This study has certain limitations. The sample size was modest (n=70), limiting power for uncommon complications. The absence of obese patients precluded assessment of body habitus as a variable influencing visibility and conversion. Additionally, a surgeon's experience could affect operative time and conversion thresholds. The effect of LPP as an added level of difficulty for trainees remains to be assessed. LPP reduces working space and visibility, making dissection and exposure technically more difficult. These limitations can temporarily steepen the learning curve for trainees, who generally perform more easily at SPP. A multicentric study with a larger sample size would enhance generalizability and strengthen the evidence base for adopting LPP as a standard approach.

## Conclusions

This study demonstrates that LPP in laparoscopic cholecystectomy is a safe and effective alternative to SPP or HPP. The findings suggest that LPP is associated with reduced postoperative pain, lower intraoperative stress, and faster gastrointestinal recovery, ultimately enhancing patient comfort and outcomes.

Notably, specific intraoperative parameters, such as elevated MAP and dilated CBD, were predictive of the need to convert to HPP, indicating that careful patient selection and intraoperative monitoring can optimize the use of LPP.

Overall, adopting LPP may offer substantial clinical benefits without compromising surgical safety or efficacy and should be considered particularly in patients in whom minimizing postoperative discomfort is a priority. Future larger-scale studies are warranted to validate these findings further and refine patient selection criteria.
